# Photosynthetic Performance of the Imidazolinone Resistant Sunflower Exposed to Single and Combined Treatment by the Herbicide Imazamox and an Amino Acid Extract

**DOI:** 10.3389/fpls.2016.01559

**Published:** 2016-10-25

**Authors:** Dobrinka A. Balabanova, Momchil Paunov, Vasillij Goltsev, Ann Cuypers, Jaco Vangronsveld, Andon Vassilev

**Affiliations:** ^1^Department of Plant Physiology and Biochemistry, Agricultural UniversityPlovdiv, Bulgaria; ^2^Centre for Environmental Sciences, Hasselt UniversityDiepenbeek, Belgium; ^3^Department of Biophysics and Radiobiology, Faculty of Biology, Sofia UniversitySofia, Bulgaria

**Keywords:** sunflower, imazamox, plant biostimulants, chlorophyll fluorescence, leaf gas exchange

## Abstract

The herbicide imazamox may provoke temporary yellowing and growth retardation in IMI-R sunflower hybrids, more often under stressful environmental conditions. Although, photosynthetic processes are not the primary sites of imazamox action, they might be influenced; therefore, more information about the photosynthetic performance of the herbicide-treated plants could be valuable for a further improvement of the Clearfield technology. Plant biostimulants have been shown to ameliorate damages caused by different stress factors on plants, but very limited information exists about their effects on herbicide-stressed plants. In order to characterize photosynthetic performance of imazamox-treated sunflower IMI-R plants, we carried out experiments including both single and combined treatments by imazamox and a plant biostimulants containing amino acid extract. We found that imazamox application in a rate of 132 μg per plant (equivalent of 40 g active ingredient ha^−1^) induced negative effects on both light-light dependent photosynthetic redox reactions and leaf gas exchange processes, which was much less pronounced after the combined application of imazamox and amino acid extract.

## Introduction

Weeds cause substantial yield losses and are one of the main limiting factors for sunflower production in Eastern Europe and the Black Sea Region, which represent more than 60% of the sunflower planted areas in the world (Kaya, 2014). To tackle this obstacle in sunflower production, a Clearfield® technology has been developed, which is based on the use of both the herbicide imazamox (imidazolinone herbicides) and resistant (IMI-R) sunflower hybrids.

Imazamox controls many annual and perennial grasses as well as broadleaf weeds. The mode of action of imazamox is inhibition of acetohydroxyacid synthase activity (AHAS, EC 2.2.1.6), also referred to as acetolactate synthase (ALS), catalyzing the first step of the branched-chain amino acids (BCAA) biosynthetic pathway. AHAS-inhibiting herbicides are a broad group, which is widely used due to their high weed control efficacy, high crop-weed selectivity, low application rates, low levels of mammalian toxicity as well as their favorable environmental profile (Shaner and Singh, 1997). However, following imazamox application, temporary yellowing and growth retardation can occur, even in IMI-R sunflower hybrids (Hanson et al., 2006; Sala et al., 2012). These effects are transient and can be more pronounced when crops are growing under stressful environmental conditions (heat, drought, waterlogged soils, etc.) (Pfenning et al., 2008).

Both yellowing and some necrosis of growth points of sunflower plants may appear days after the treatment, but profound changes in their metabolism occur soon after the herbicide application (Tan et al., 2005). Although, the photosynthetic process is not a primary target of imidazolinone herbicides, changes in photosynthetic performance have been detected in different crops after application. For example, the rate of CO_2_ fixation was reduced in imazamox-treated wheat plants, but to a different extent in the tested Clearfield cultivars (Jimenez et al., 2015). An initial, but reversible damage to the photosynthetic apparatus of the Clearfield rice cultivar Puitá Inta CL exposed to imidazolinone herbicides was observed by use of the chlorophyll fluorescence JIP test (Sousa et al., 2013). Other authors did not find significant effects on photosynthetic electron transport in cucumber cotyledons, treated by imidazolinone herbicides (Dayan and Zaccaro, 2012). Obviously, the appearance and gravity of the observed physiological disorders in crops depends on many factors—different herbicide retention on the leaf surface, impaired uptake, reduced translocation, herbicide detoxification as well as insensitivity of the target enzyme to the herbicide (Tan et al., 2005; Yu and Powles, 2014).

Photosynthesis and photosynthesis-related parameters, such as chlorophyll fluorescence are recognized as good indicators of herbicide induced injury (Dayan and Zaccaro, 2012). Even though photosynthetic processes are not the primary sites of herbicidal action, some impact may be expected due to possible feedback inhibition. The measurement of leaf gas exchange and chlorophyll fluorescence are fast and non-destructive allowing kinetic monitoring of the plant physiological status; therefore, more information about the photosynthetic responses of the sunflower Clearfield genotypes to imazamox could be valuable to further improve their selectivity and resistance.

The performance of plants exposed to different stress factors, including herbicides, could be improved by the use of a new group of agricultural products called biostimulants (Calvo et al., 2014). A plant biostimulant is any substance or microorganism applied to plants with the aim to improve nutrition efficiency, abiotic stress tolerance and/or crop quality traits, regardless of its nutrients content (du Jardin, 2015). Biostimulants are a diverse group containing different compounds and substances, including also protein hydrolysates. Protein hydrolysates (PHs) are defined as mixtures of polypeptides, oligopeptides and amino acids that are manufactured from protein sources using partial hydrolysis (Schaafsma, 2009). The application of PHs has been shown to avoid or reduce production losses caused by unfavorable soil conditions and environmental stresses, such as temperature, drought, salinity and others (Botta, 2013; Petrozza et al., 2014; Lucini et al., 2015). Surprisingly, there exists very limited information about the effects of protein hydrolysates on herbicide-stressed plants. The only report we found showed a small, but not significant yield increase of oat and winter wheat after addition of biostimulants to the used post emergence herbicides (Soltani et al., 2015).

The primary aim of our study was to describe the functional status of the photosynthetic apparatus of imazamox-treated IMI-R sunflower plants. Considering the lack of information concerning the effects of protein hydrolysates against herbicide stress, the second aim of the study was to explore whether the application of PHs-based biostimulants could improve photosynthetic performance and growth of these plants.

## Materials and methods

### Plant material and treatment

The sunflower (*Helianthus annuus*) Clearfield® hybrid Mildimi carrying the haplotype 5 of the AHAS1 gene (*Imisun* trait) was used in this study. Seeds were washed with distilled water and germinated in Petri dishes for 3 days at 22°C. The seedlings were placed in 2.5 L pots (4 plants per pot) filled with nutrient solution containing: 0.505 mM KNO_3_, 0.15 mM Ca(NO_3_)_2_ × 4H_2_O, 0.1 mM NH_4_H_2_PO_4_, 0.1 mM MgSO_4_ × 7H_2_O, 4.63 mM H_3_BO_3_, 0.91 mM MnCl_2_ × 4H_2_O, 0.03 mM CuSO_4_ × 5H_2_O, 0.06 mM H_2_MoO_4_ × H_2_O, 0.16 mM ZnSO_4_ × 7H_2_O, 1.64 mM FeSO_4_ × 7H_2_O, and 0.81 mM Na_2_-EDTA. The plants were grown in a growth chamber at controlled environmental conditions: photoperiod 14/10 h (light/dark), 250 μmol m^−2^ s^−1^ photosynthetic photon flux density at leaf level, temperature 24/22 ± 1C° day/night and 60–65% relative air humidity. The pH of the nutrient solution was 5.8 ± 0.1. It was aerated continuously and refreshed within each 3 days.

The experimental design including 4 treatments, was set up at 3rd pair leaves old plants, namely:

1. Non-treated plants (control);
2. Foliar application of amino acid extract (AAE) (Terra-sorb foliar, 10 μl per plant, equivalent to 3 L ha^−1^), a biostimulant based on PHs, produced by Bioiberica S.A., Spain, which was obtained from selected animal tissues by enzymatic hydrolysis. The aminogram of the product is presented in the Supplementary Files.
3. Foliar application of imazamox (132 μg per plant, equivalent of 40 g active ingredient ha^−1^), a herbicide with trade name Pulsar 40, produced by BASF chemical company and
4. Combined application of imazamox and AAE.

After the application, the plants were kept two more weeks at the same growing conditions. The entire experiment was repeated twice.

### Plant growth measurements

Fresh weight and height of the plants as well as their leaf area were determined at the end of the experimental period. Ten plants were used for each treatment. The leaf area was measured by an electronic areameter (NEO-2, TU-Sofia, Bulgaria).

### Imazamox residues determination

Leaf samples (10 g) were collected 7 days after treatment (DAT), washed by distilled water and stored at −86°C until analysis. The further sample procedure included homogenization and extraction by 30 ml acetone, followed by 30 ml mixture of petroleum ether/dichloromethane (1/1) added to the same solution. Subsequently, 20 g of NaSO_4_ were added, followed by 30 min incubation at room temperature. Fifteen ml from the solvent were evaporated to dryness on a rotary evaporator at 40°C bath temperature and the residues were dissolved in 10 ml methanol/water (1/1) mixture.

The imazamox residues were analyzed by liquid chromatography LC-MS/MS tandem quadrupole mass spectrometer Acquity XevoTQ UPLS/MS/MS, from Waters (Waters, USA), using a column SunFire, C_18_, 2.1 × 150 mm, 3.5 μm, combined with a pre-column SunFire, C_18_, 2.1 × 10 mm, 3.5 μm, both from Waters (Waters, USA). LC-MS ion scans for the m/z 306.1 in positive ion mode were performed at a cone voltage of 35 V and a collision energy of 20 eV (1st transition m/z 261,05) and collision energy of 27 eV (2nd transition m/z 85,95). The obtained results were expressed in mg kg^−1^ FW.

### Leaf gas exchange analysis

Leaf gas exchange (net photosynthetic rate—A, transpiration rate—E, stomatal conductance - gs and internal CO_2_ concentration—c_i_) was measured on the fully developed leaves (closest to the top) at the end of the experimental period with an open photosynthetic system LCpro+ (Analytical Development Company Ltd., Hoddesdon, England), equipped with a broad chamber. The conditions during the measurements were: light intensity—250 μmol m^−1^ s^−1^ (PAR), CO_2_ concentration—350 μmol mol^−1^, leaf temperature 24–25°C, relative humidity—60–65%. The net photosynthetic rate (A) was determined based on the decrease of the CO_2_ concentration in the chamber. The transpiration rate (E) was determined based the increasing concentration of water vapor. The stomatal conductance (gs) was determined automatically by using the records for E, temperature, energy balance in the chamber and the water vapor concentration.

### Photosynthetic pigments content

Photosynthetic pigments (chlorophyll *a*, chlorophyll *b* and total carotenoids) were extracted in 80% (v/v) acetone, measured spectrophotometrically and calculated according to the formulae of Lichtenthaler (1987).

### Chlorophyll fluorescence analysis

Chlorophyll fluorescence (ChlF) analysis was performed using a Handy PEA fluorimeter (Handy Plant Efficiency Analyzer, Hansatech Instruments Ltd., King's Lynn, UK) on native leaves of plants at 7 DAT. The measured spots of the leaves were dark-adapted for 1 h while the plants were left in light. Induction curves of ChlF were recorded for 1 s with 3000 μmol m^−2^ s^−1^ PPFD. For each experimental treatment at least 10 measurements were performed. The primary data processing was done using the HandyBarley program, developed by Petko Chernev at the Department of Biophysics and Radiobiology, Faculty of Biology, Sofia University, and the secondary processing, including calculation of JIP parameters—on Microsoft Excel. The plots were made in Sigma Plot.

The intensity of the ChlF was recorded in arbitrary units. Those were transformed into relative units of the relative variable fluorescence (Vt) by double normalization to F_O_ and F_M_. When the Vt values of the untreated control were subtracted from the values of the other treatments at the corresponding moment in the induction time differential curves were built. Such curves were made also after double normalization from F_O_ to F_J_ and F_O_ to F_K_ (at 0.3 ms).

The fluorescence intensities determined at 50 μs, 100 μs, 300 μs, 2 ms, 30 ms, and F_M_ were used for the calculation of the OJIP test parameters (Strasser et al., 1995, 2004, 2010; Tsimilli-Michael and Strasser, 2008; Stirbet and Govindjee, 2011; Kalaji et al., 2014) that are presented on Table 1.

**Table 1:** **Summary of measured and calculated Chl ***a*** fluorescence parameters**.

**Fluorescence parameter**	**Description**
**MEASURED PARAMETERS AND BASIC JIP-TEST PARAMETERS DERIVED FROM THE OJIP TRANSIENT**	
F_O_ = F_20μs_	Minimum fluorescence, when all PSII reaction centers (RCs) are open, Fluorescence intensity at 20 μs
F_J_ = F_2ms_	Fluorescence intensity at the J-step (2 ms)
F_I_ = F_30ms_	Fluorescence intensity at the I-step (30 ms)
F_M_ = F_P_	Maximum recorded fluorescence, when all PSII RCs are closed
S_M_ = A_M_/(F_M_ − F_O_), where A_M_ is the area above the OJIP curve between F_O_ and F_M_ and the F_M_ asymptote	Standardized area above the fluorescence curve between F_O_ and F_M_ is proportional to the pool size of the electron acceptors on the reducing side of Photosystem II
V_J_ = (F_2ms_− F_O_)/(F_M_ – F_O_)	Relative variable fluorescence at J-step (2 ms)
M_0_ = 4 (F_300μ*s*_ − F_O_)/(F_M_ – F_O_)	Approximated initial slope of the fluorescent transient. This parameter is related to rate of closure of reaction centers
**SPECIFIC ENERGY FLUXES EXPRESSED PER ACTIVE PSII REACTION CENTER (RC)**	
ABS/RC = M_0_ × (1/V_J_) × [1 − (F_O_/F_M_)]	Apparent antenna size of active PSII RC
TR_0_/RC = M_0_ × (1/V_J_)	Trapping flux leading to Q_A_ reduction per RC
ET_0_/RC = M_0_^*^(1/V_J_)^*^ψ0, where ψ0 = (1 − V_J_)	Electron transport flux per reaction center (RC) at *t* = 0
RE_0_/RC = M_0_(1/V_J_)(1 − V_J_)	Quantum yield of electron transport from ${\text{Q}}_{\text{A}}^{-}$ to the PSI end electron acceptors
RC/CS_0_	Number of active PSII RCs per illuminated cross-section (CS) at initial moment of illumination (at *t* = 0)
DI_0_/R_C_ = (ABS/RC) − (TR_0_/RC)	Dissipated energy flux per reaction center (RC) at *t* = 0
N = (S_M_/S_*S*_), where S_*S*_ = V_J_/M_0_	Number indicating how many times Q_A_ is reduced while fluorescence reaches its maximal value (number of Q_A_ redox turnovers until F_M_ is reached); S_S_, normalized curve above O-J curve.
**Qantum yields and probabilities**	
φ_Po_ ≡ TR_0_/ABS = [1 − F_O_/F_M_)] = F_V_/F_M_	Maximum quantum yield of primary PSII photochemistry
φ_*Eo*_ = (1 − F_J_/F_M_)(1 − V_J_)	Quantum yield for electron transport from ${\text{Q}}_{\text{A}}^{-}$ to plastoquinone
φ_Ro_ = (1 − F_I_/F_M_)(1 − V_J_)	Quantum yield for reduction of end electron acceptors at the PSI acceptor side (RE)
γRC	Probability, that PSII chlorophyll molecule function as RC
**PERFORMANCE INDEXES AND DRIVING FORCES**	
PI_ABS_ = γRC/(1 −γRC) × φ_Po_/(1 −φ_Po_) × ψo/(1 −ψo)	Performance index of electron flux from PSII based to intersystem acceptors
PI_total_ = PI_ABS_ × δ_Ro_/(1 – δ_Ro_), where δ_Ro_ = (1 − V_J_)/(1 − V_I_)	Performance index of electron flux to the final PSI electron acceptors

*Based on (Strasser et al., 1995, 2004, 2010; Tsimilli-Michael and Strasser, 2008; Stirbet and Govindjee, 2011; Kalaji et al., 2014)*.

### Statistical analysis

Statistical analysis was performed using one way ANOVA (for *P* < 0.05). Based on ANOVA results, a Duncan test for mean comparison was performed, for a 95% confidence level, to test for significant differences among treatments. In the figures, different letters (a, b, c) express significant differences.

## Results

### Plant performance and growth response

The recommended field dose of imazamox for IMI-resistant sunflower hybrids is 48 g active ingredient ha^−1^. Considering that plants cultivated in a growth chamber could have a higher sensitivity to different stress factors as compared to those grown in the field, we performed a range-finding study to identify a suitable imazamox dose able to induce chronic herbicidal stress in growth chamber cultivated sunflower plants. The selected dose was 132 μg per sunflower plant, which is equal to 40 g active ingredient ha^−1^. Sunflower plants receiving this imazamox dose showed obvious toxicity symptoms.

Both, leaf chlorosis and deformations in young leaves developed in imazamox-treated sunflower plants. These symptoms were strongly pronounced at 7 DAT, when small necrotic spots appeared in the most injured leaves. At 14 DAT, the plants developed new leaves without visual symptoms of toxicity, but the latter subsisted in the older leaves of the sunflower plants.

**Figure 1:**
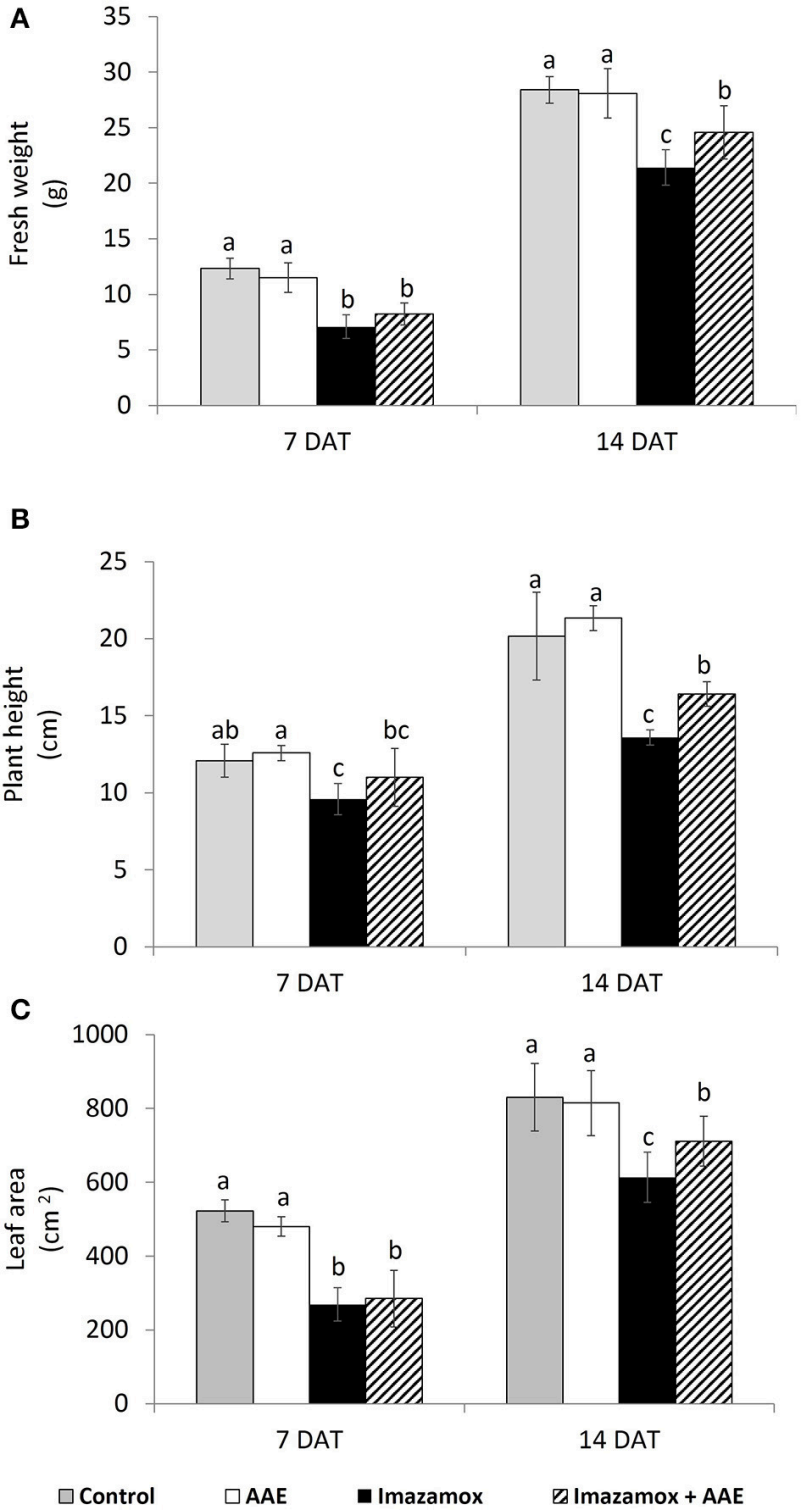
**Growth parameters [(A) fresh weight; (B) plant height; (C) leaf area] of imidazolinone resistant sunflower plants, exposed to single and combined treatment by imazamox and AAE**. The values represent the mean of three biological replicates. Different letters (a, b, c) express significant differences (*P* < 0.05).

Imazamox-treated plants were characterized by delayed growth. The growth inhibition was significant at 7 DAT with 42.5, 29.6, and 48.4% decreased fresh weight, length and leaf area, respectively, in comparison to the untreated plants (Figure 1). At 14 DAT the growth inhibition in imazamox-treated sunflower plants was still significant, but less pronounced. Application of only amino acid extract (AAE) did not have any effects on sunflower plants at 7 and 14 DAT, but the growth of plants exposed to the combined treatment (AAE + imazamox) was less retarded and their performance was better as compared with that of imazamox-treated plants. This effect was limited at 7 DAT and more pronounced at 14 DAT.

### Leaf imazamox residues

The resistance of IMI-R sunflower plants to imazamox is due to many factors; one of them is an enhanced degradation rate. In our study, the residual herbicide concentration in the leaves of imazamox-treated plants was 6.86 ± 0.31 mg kg^−1^ at 7 DAT. The imazamox level in the leaves of plants exposed to the combined treatment of AAE and imazamox was quite similar (7.11 ± 0.23 mg kg^−1^) providing evidence that the both compounds did not interfere during the infiltration process. In fact, some remainings of both herbicide and AAE on external leaf surface cannot be excluded.

### Leaf gas exchange

**Figure 2:**
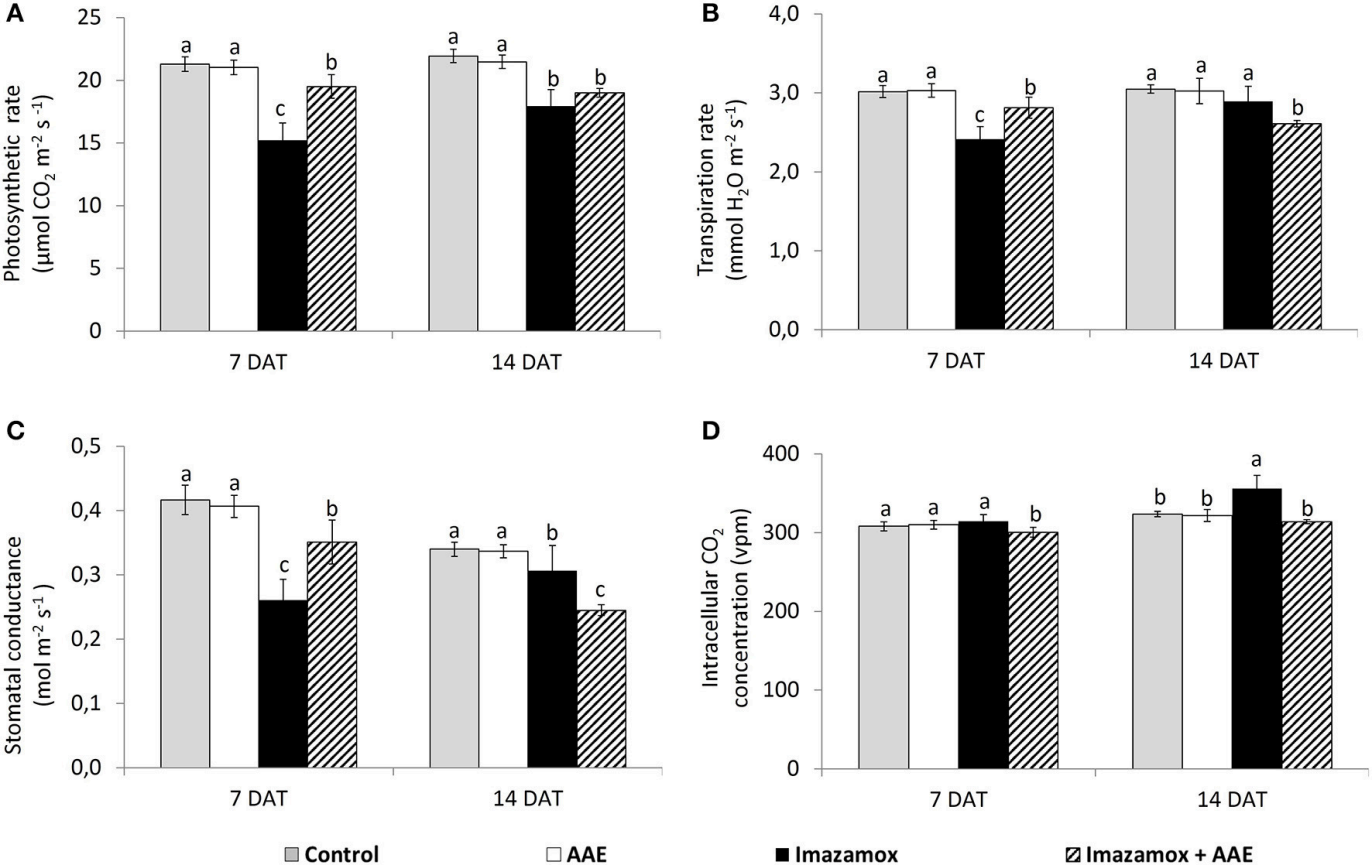
**Leaf gas exchange parameters [(A) net photosynthetic rate; (B) transpiration rate; (C) stomatal condictance; (D) intracellular CO_**2**_ concentration] of imidazolinone resistant sunflower plants, exposed to single and combined treatment by imazamox and AAE**. The values represent the mean of three biological replicates. Different letters (a, b, c) express significant differences (*P* < 0.05).

The application of imazamox caused significant decreases of the leaf gas exchange parameters in the sunflower plants at 7 DAT (Figure 2). The net photosynthetic rate (A) in imazamox-treated plants diminished by 28.7%, transpiration rate (E) and stomatal conductance (gs) by respectively 20.0 and 37.3%. The leaf gas exchange parameters slightly recovered at 14 DAT and the differences between treated and untreated plants at 14 DAT were smaller than at 7 DAT. While the recovery of E was almost complete, the inhibition of A still was 19.9 % lower than in untreated control plants.

Application of only AAE did not have any effect on leaf gas exchange parameters of sunflower plants, but adding of AAE to the imazamox solution (combined treatment) resulted in a slight improvement of plant performance in comparison to just imazamox. At 7 DAT the A of plants treated with imazamox + AAE was less inhibited (8.4%) than that of plants treated with only imazamox (28.7%) in comparison to untreated control plants. Such a tendency was still observed at 14 DAT, the respective A values were 13.4 and 18.5% lower than those of the untreated controls. The changes in E and gs were similar to that of A at 7 DAT. This improvement of photosynthetic performance was not related to the changes in internal concentration of CO_2_, which was not significantly different from that of the control plants.

### Photosynthetic pigments profiling

**Figure 3:**
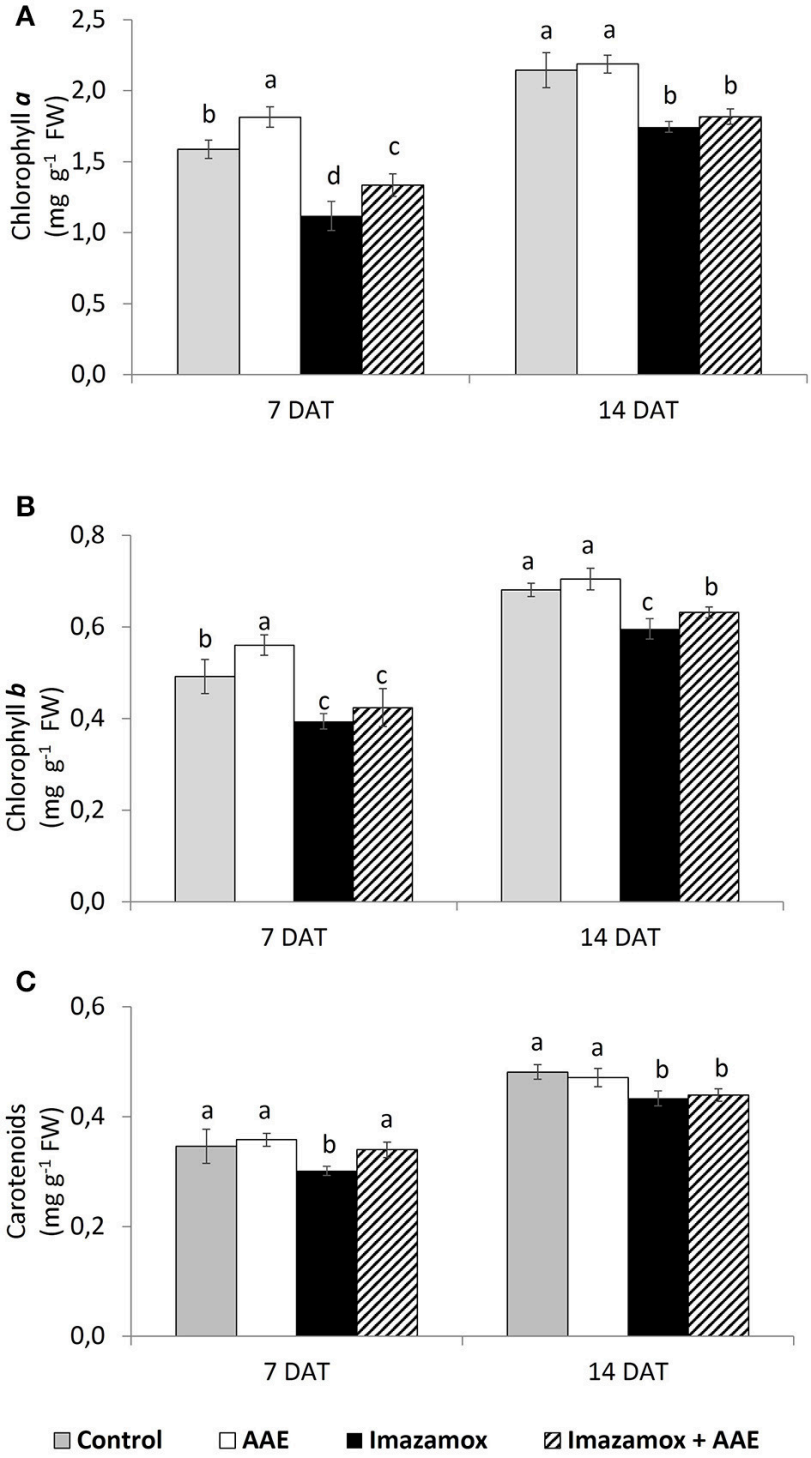
**Content of photosynthetic pigments [(A) chlorophyll ***a***; (B) chlorophyll ***b***; (C)—total carotenoids] in imidazolinine resistant sunflower plants, exposed to single and combined treatments by imazamox and AAE**. The values represent the mean of three biological replicates. Different letters (a, b, c) express significant differences (*P* < 0.05).

Application of imazamox significantly decreased the contents of chlorophyll *a* (chl *a*), chlorophyll *b* (chl *b*) and total carotenoids (car) (Figure 3). The concentrations of chl *a* and chl *b* at 7 DAT were respectively 29.5 and 19.8% lower, while there were 12.9 % less car. in comparison to the untreated control. This effect of the imazamox diminished in time and the differences in photosynthetic pigments content between imazamox-treated plants and untreated controls were smaller at 14 DAT—from 18.6% for chl *a* to 8.7% for car.

The application of AAE only significantly increased the total chlorophyll content by an average of 13%. At 7 DAT the positive effect of AAE on the photosynthetic pigments was also observed in plants exposed to the combination of imazamox + AAE: in these plants, the levels of chl *a* and chl *b* as well as that of car were diminished less than those in plants that received only imazamox. The respective values in percent were 15.8, 13.8, and 1.8%.

### Chlorophyll fluorescence

**Figure 4:**
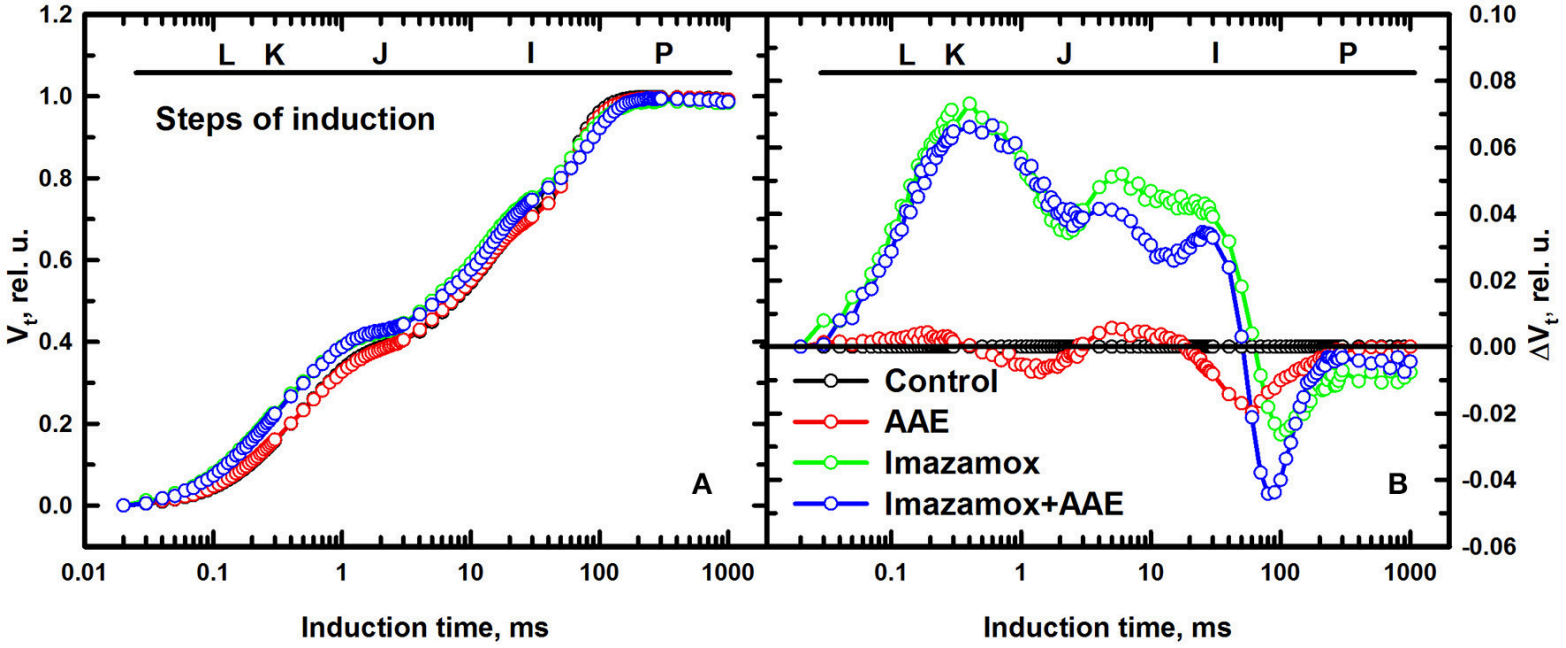
**(A)** Relative variable fluorescence (Vt) transients recorded for 1 s with 3000 μmol m^**−2**^ s^**−1**^ PPFD after 1 h dark-adaptation of the measured spots on native leaves of imidazolinone resistant sunflower plants, exposed to single and combined treatment by imazamox and AAE, 7 DAT. Non-treated plants were used as controls. **(B)** Differential curves of relative variable fluorescence when the Vt values of ChlF rise recorded in control plants is subtracted from the corresponding values measured in treated plants.

The data presented in Figure 4A describing one-second induction transients of the relative variable fluorescence showed slight differences between the different treatments at 7 DAT. The transients show the typical steps of induction of ChlF: O, initial fluorescence level; J, is recorded when the rates of reduction and oxidation of Q_A_ become equal (2 ms after starting of illumination); I, recorded at 30 ms when the rate of reduction and oxidation of plastoquinone (PQ) are equal; P, maximal ChlF level, recorded at 300 ms when the PQ pool is fully reduced. Though the overall shape of the rise was highly similar for the different treatments, the steps of induction J and I were different.

To visualize and analyze those differences throughout the induction time, the differential curves (Oukarroum et al., 2007) were calculated by subtracting the Vt curve of the untreated control from the curves recorded for the treated plants (Figure 4B). The single imazamox and combined imazamox + AAE treatments showed positive ΔVt values from O until the induction transient I-P, where the ΔVt values turned negative until P (zero by definition). Moreover, the progress of both curves is very similar from O to J while different from J to I. Positive ΔVt values indicate lower rates, i.e., decreased efficiency of electron transport and negative values the opposite. The AAE treatment showed a fluorescence transient close to that of the untreated control. These findings indicate that imazamox had a prolific inhibition effect on the light phase photosynthetic reactions even if its specific site of action is not photosynthesis while the biostimulant altered them just slightly. In addition, when added together with imazamox, a slight beneficial effect of AAE was indicated by the lower ΔVt values during J-I transient in comparison to the single herbicide treatment.

**Figure 5:**
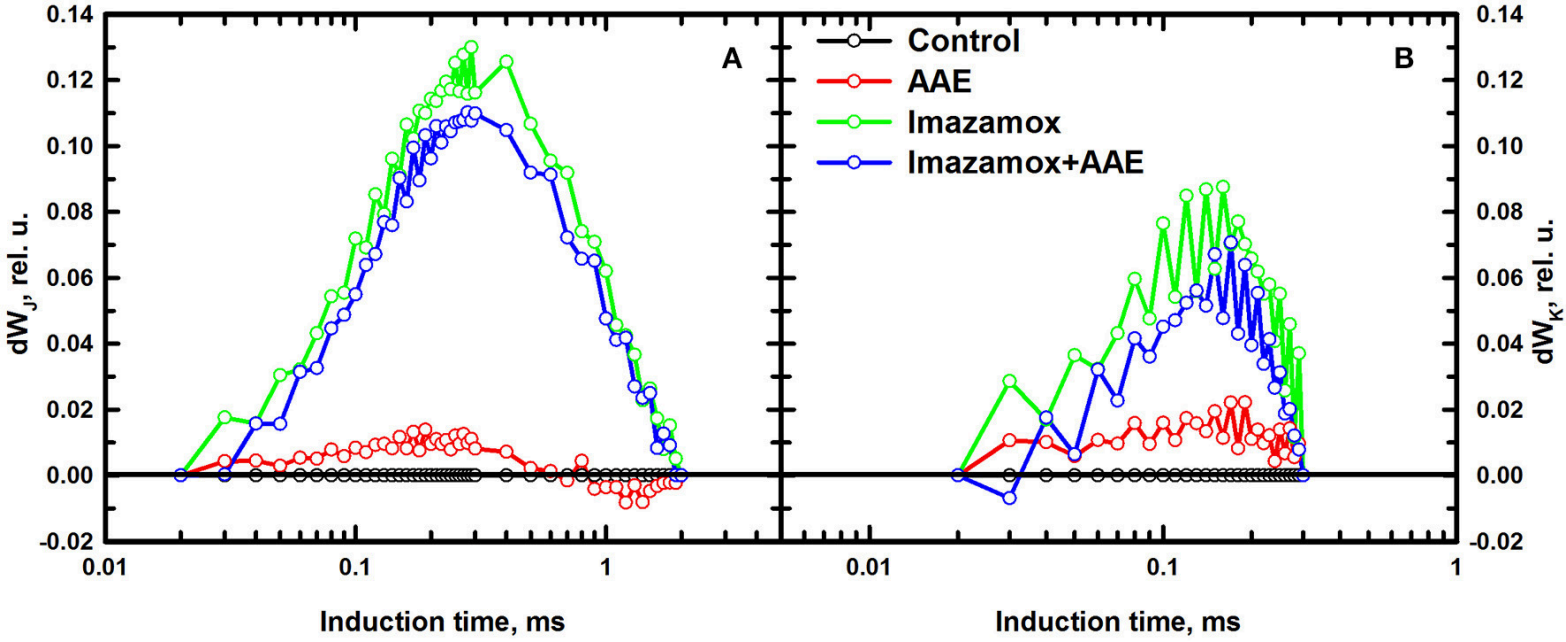
**Differential curves of relative variable fluorescence, double normalized from FO to FJ (A) and from FO to FK (B), acquired from native leaves of imizadolinone resistant sunflower plants, exposed to single and combined treatment by imazamox and AAE, 7 DAT**. Non-treated plants were used as controls. Experimental conditions are the same as in Figure 4.

The differential curves composed from O to J (Figure 5A) provide information about the balance of the electron transport through PSII. A pronounced positive peak at K was observed when imazamox was added alone or together with AAE. The positive K peak which is a sign for disturbances in the oxygen evolving complex is often observed during stress conditions (Strasser et al., 2004). The differential curves constructed from O to K (Figure 5B) are associated with the level of energy transfer between antennae complexes of different RC, i.e., photosynthetic unit connectivity. Positive values at 0.1 ms are known as L band and indicate lower connectivity as was the case for the imazamox treatment.

**Figure 6:**
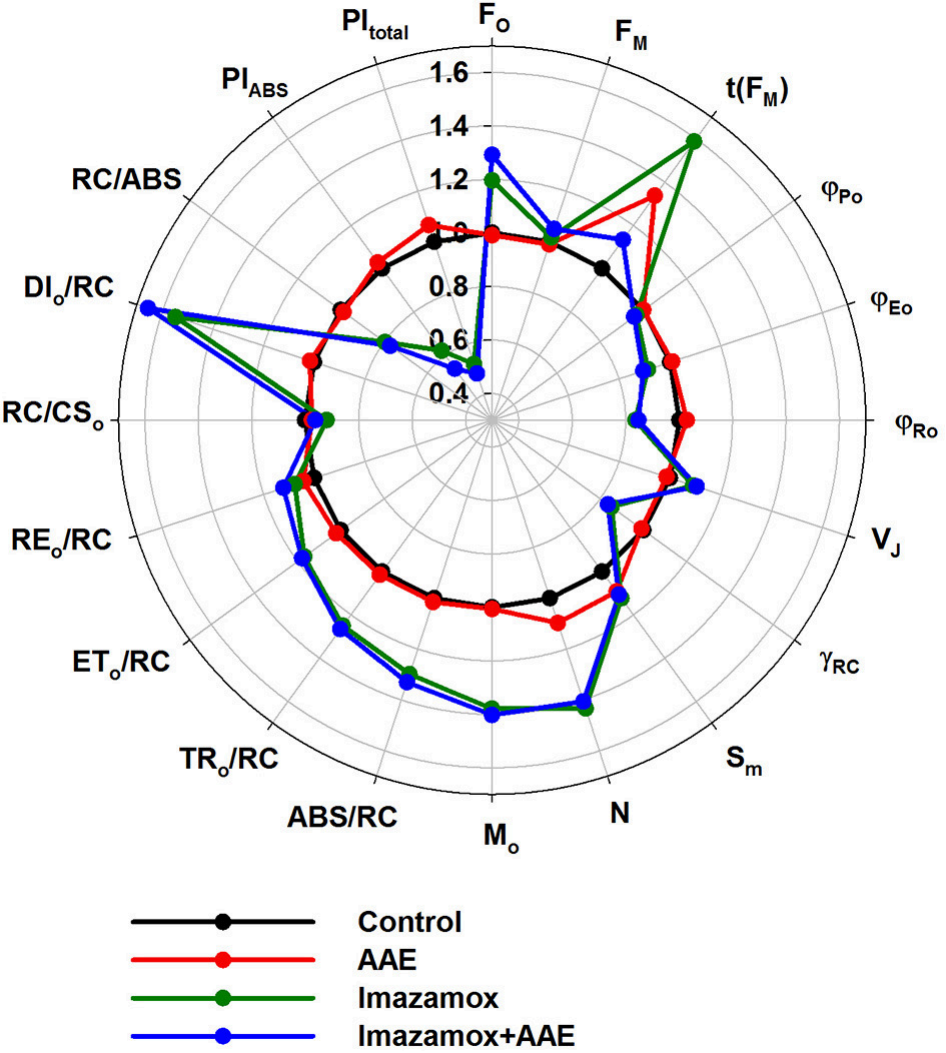
**OJIP test parameters derived from ChlF induction transients recorded from native leaves of imidazolinone resistant sunflower plants, exposed to single and combined treatment by imazamox and AAE, 7 DAT**. Non-treated plants were used as controls. Experimental conditions are the same as in Figure 4.

OJIP test parameters were calculated from the ChlF transients (Figure 6). Once again the effect of the imazamox was obvious. Application of imazamox lead to higher F_O_, M_O_, ABS/RC, lower γ_RC_ and the almost unchanged RC/CS_0_ indicate more chlorophyll *a* pigments in the antenna that could not transfer their energy to a RC and thus emit fluorescence. This phenomenon can be attributed to the lowered φ(Po) and elevated DI_O_/RC, i.e., rise in the photochemically inactive PSII RCs. In addition, the increases of parameters t(F_M_), S_M_ and N after imazamox treatment indicate increased relative numbers of electron acceptors in the PQ pool or at the PSI acceptor side per RC. We hypothesize that these observations are due to a decreased *de novo* synthesis of reaction center proteins as a result of the inhibitory effect of imazamox on the branched amino acid synthesis.

φ_Eo_ and φ_Ro_ got lower in the imazamox-treated plants in correspondence to the higher ChlF values at J and I, resulting in decreases of both the PI_ABS_ and PI_total_. These parameters summarize the fact that the efficiency of the photosynthetic light phase was negatively impacted by imazamox. As for the AAE action, the overall picture of the OJIP test parameters indicates that it did not alter the imazamox effect as well as the state of the photosynthetic machinery in the untreated control plants.

## Discussion

Imazamox is readily absorbed by leaves (Shaner and O'Connor, 1991) and subsequently translocated through the phloem and xylem to the meristems of plants (Shaner, 2003). In resistant species, imazamox metabolism occurs by oxidative hydroxylation on the pyridine ring, followed by carbohydrate conjugation (Ohba et al., 1997). When imazamox penetrating through the leaf cuticle of crop species is not degraded to a sufficiently low level, it may induce different toxicity effects. Appearance of leaf chlorosis is one of the most typical symptoms caused by imidazolinone herbicide treatment (Ochogavía et al., 2014). Because of the high sensitivity of meristematic tissues, one of the first visible effects of AHAS-inhibitors is growth inhibition (Shaner and Mallipudi, 1991). The chlorosis and partial deformation of younger leaves that we observed in imazamox-treated sunflower plants correspond with what other authors reported (Sala et al., 2012). The appearance of chlorosis in the leaves of imazamox-treated sunflower plants in our study was due to their significantly lower chlorophyll content (Figure 3). This observation is in accordance with the data presented by Alonge (2000) for the decreased chlorophyll *a* content in imazaquin-treated soybean plants as well as imidazolinine herbicide treated sunflower and wheat plants (Pozniak et al., 2004; Ochogavía et al., 2014).

The photosynthetic performance of crop plants is very sensitive to stress factors, including herbicides. The herbicides may influence directly or indirectly different sub-processes of photosynthesis; stomatal conductance, synthesis/degradation of photosynthetic pigments, light dependent processes, Calvin cycle reactions, transport of photoassimilates, etc. Evidence for such negative effects was found also in our study. The negative impact of imazamox on net photosynthetic rate (A) of sunflower plants at 7 DAT (Figure 2) was partly due to decreased stomatal conductance to CO_2_ uptake, lowered chlorophyll content as well as electron transport processes in thylakoid membranes. These results correspond with those of Gaston et al. (2002), who reported significant A inhibition in imazethapyr-treated pea plants 7 DAT. Generally, A depends on stomatal and non-stomatal factors and both of them were reported as limiting factors in imidazolinone herbicide-treated plants. Anastasov (2010) described a reduction of the number of stomata in sunflower plants after imazamox application. Also decreased utilization of carbohydrates in imidazolinone herbicides treated *Arabidopsis* and pea plants was found (Zabalza et al., 2004; Qian et al., 2011), which might be attributed to a decreased sink strength (Zabalza et al., 2004). The time-course measurements of photosynthetic performance of imazamox-treated sunflower plants (7 and 14 DAT) revealed a tendency to recover, which could be explained by degradation and/or detoxification of the herbicide as well as expression of different defense mechanisms. Such a tendency was also reported by Jimenez et al. (2015) in IMI-resistant wheat cultivars.

In addition to the results confirming the negative impacts of imazamox on sunflower plants, which could be consequences of high rate application, unfavorable climatic conditions or differences in genotype selectivity, etc. our study describes in more detail the herbicide effects on light dependent photosynthetic processes. Using a sensitive method, based on high time-resolution measurements of the fast photoinduced changes of chl *a* fluorescence emitted mainly by antennae pigments of PSII (Papageorgiou and Govindjee, 2004; Stirbet and Govindjee, 2011), we detected some specific aspects of imazamox-photosynthesis interactions related to both structure and function of the photosynthetic machinery.

Imazamox clearly affects different light dependent photosynthetic redox reactions. We found that the concentration of active PSII reaction centers (RC/CS_0_) in imazamox-treated plants was slightly diminished, but considering the significant increase of the total chlorophyll concentration in the leaves during the period of investigation (14 days), the relative part of active reaction centers within the total chlorophyll content (represented by parameter γ_RC_) significantly decreased. This leads to an increase of the relative antennae size of each active RC (ABS/RC) and the number of the photoinduced turnovers of PSII RCs required for full reduction of photosynthetic electron transport chain (N). The structural and functional interaction between antennae complexes of PSII was disturbed as a result of imazamox treatment, which is monitored by the change of fluorescence rise dymamics within the first 300 μs of illumination (Figure 5B). Both, the performance of the photosynthetic light phase as a whole and of the reactions in PSII (monitored by parameters PI_total_ and PI_ASB_, respectively) decreased.

The accumulation of imazamox in the meristematic tissues of sunflower plants together with the negatively impacted photosynthetic performance resulted in growth retardation, which agrees with the data of other authors, reporting inhibited growth after AHAS herbicide treatment in IMI-R crops, such as wheat and bean (Hanson et al., 2006; García-Garijo et al., 2014).

The observed and detected positive effects of the foliar-applied biostimulant on both growth and performance of imazamox-treated sunflower plants could be due to different reasons. Watson and Fowden (1975) and more recently by Matsumiya and Kubo (2011) demonstrated that both amino acids and small peptides present in PHs biosimulant are absorbed by leaves. It also has been shown that the growth retardation induced by AHAS inhibiting herbicides may be overcome by the application of BCAA (Cobb and Reade, 2010). Ertani et al. (2009) reported that the application of PHs stimulates nitrogen assimilation in plants. Therefore, we may speculate that the application of amino acid extract might compensate to some extent herbicide-induced deficiency of BCAA by supporting the plants' protein turnover. Many other processes of secondary plant metabolism such as stimulation of flavonoid, terpenes and glucosinolates biosynthesis have also been reported to be positively affected by PHs and hereby increasing their defense responses and tolerance against stresses (Ertani et al., 2011; Colla et al., 2015; Lucini et al., 2015).

In conclusion, in our experimental conditions, the herbicide imazamox caused a transient inhibition of the photosynthetic performance and growth of sunflower IMI-R plants from the hybrid Mildimi. The negative impact is obvious on both light-light dependent photosynthetic redox reactions and leaf gas exchange processes. Combined application of imazamox and the amino acid extract diminished the negative effects of the herbicide, but further studies are needed to clarify the nature and mechanisms of the protective effect(s) of the biostumulant.

## Author contributions

Conceived and designed the experiments: AV, AC, and JV. Performed the experiments: DB. Analyzed the data: DB, MP, and VG. Prepared the manuscript: DB, VG, AV, AC, and JV. Revised the manuscript: JV.

## Funding

This work is funded by an UHasselt BOF-BILA grant to DB in the frame of a collaboration between Hasselt University, Belgium and Agricultural University of Plovdiv, Bulgaria. The work was further supported by the Methusalem project 08M03VGRJ and JV.

### Conflict of interest statement

The authors declare that the research was conducted in the absence of any commercial or financial relationships that could be construed as a potential conflict of interest.
