# Supplementary material for: Photosynthetic Performance of the Imidazolinone Resistant Sunflower Exposed to Single and Combined Treatment by the Herbicide Imazamox and an Amino Acid Extract
Source: Front Plant Sci. 2016 Oct 25;7:1559. doi: 10.3389/fpls.2016.01559 (PMC5078751; doi:10.3389/fpls.2016.01559)
Supplement: Supplementary file 1 [file DataSheet1.PDF]

TYPICAL AMINOGRAM

Terra-Sorb® Foliar

FREE AMINO ACIDS	%
Aspartic Acid	0.57
Serine	0.40
Glutamic Acid	4.25
Glycine	1.86
Histidine	0.15
Arginine	0.11
Threonine	0.20
Alanine	0.62
Proline	0.35
Cisteine	0.05
Tyrosine	0.55
Valina	0.10
Methionine	0.15
Lysina	0.57
Isoleucine	0.31
Leucine	0.55
Phenylalanine	0.21
Tryptophane	0.10
Total	11.10
Total guaranteed in label	9.30
