# Supplementary material for: Photosynthetic Performance of the Imidazolinone Resistant Sunflower Exposed to Single and Combined Treatment by the Herbicide Imazamox and an Amino Acid Extract
Source: Front Plant Sci. 2016 Oct 25;7:1559. doi: 10.3389/fpls.2016.01559 (PMC5078751; doi:10.3389/fpls.2016.01559)
Supplement: Supplementary file 2 [file DataSheet2.PDF]

BIOIBERICA

CERTIFICATE OF ANALYSIS

TERRA-SORB FOLIAR

ORGANOLEPTIC CHARACTERITICS	:	Green Liquid
pH	:	5.2
DENSITY	:	1.09 g/ml

CHEMICAL COMPOSITION

FREE AMINO ACIDS	:	9.3 % w/w
TOTAL NITROGEN	:	2.1 %w/w
BORON	:	0.02 % w/w
MANGANESE	:	0.046 % w/w
ZINC	:	0.067 % w/w
